# Dynamic Scheduling of Contextually Categorised Internet of Things Services in Fog Computing Environment

**DOI:** 10.3390/s22020465

**Published:** 2022-01-08

**Authors:** Petar Krivic, Mario Kusek, Igor Cavrak, Pavle Skocir

**Affiliations:** Faculty of Electrical Engineering and Computing, University of Zagreb, 10000 Zagreb, Croatia; mario.kusek@fer.hr (M.K.); igor.cavrak@fer.hr (I.C.); pavle.skocir@fer.hr (P.S.)

**Keywords:** fog computing, Internet of Things (IoT), context-aware systems, service scheduling, service categorization, smart environments, service orchestration, QoS

## Abstract

Fog computing emerged as a concept that responds to the requirements of upcoming solutions requiring optimizations primarily in the context of the following QoS parameters: latency, throughput, reliability, security, and network traffic reduction. The rapid development of local computing devices and container-based virtualization enabled the application of fog computing within the IoT environment. However, it is necessary to utilize algorithm-based service scheduling that considers the targeted QoS parameters to optimize the service performance and reach the potential of the fog computing concept. In this paper, we first describe our categorization of IoT services that affects the execution of our scheduling algorithm. Secondly, we propose our scheduling algorithm that considers the context of processing devices, user context, and service context to determine the optimal schedule for the execution of service components across the distributed fog-to-cloud environment. The conducted simulations confirmed the performance of the proposed algorithm and showcased its major contribution—dynamic scheduling, i.e., the responsiveness to the volatile QoS parameters due to changeable network conditions. Thus, we successfully demonstrated that our dynamic scheduling algorithm enhances the efficiency of service performance based on the targeted QoS criteria of the specific service scenario.

## 1. Introduction

The concept of fog computing emerged as a novel architecture that could improve the efficiency of service delivery depending on the attributes of the specific service scenario, particularly in the context of latency, reliability, security, and network traffic reduction [1,2]. The extensive development of broadly available computing devices and the perpetual need to optimize the general efficiency in service delivery resulted in the idea to utilize local processing capacities in addition to the commonly used centralized cloud processing model. Offloading a certain amount of processing to the local environments reduces the amount of traffic in the public network and eliminates the propagation latency by default. Still, the realization of distributed processing presents a complex challenge that is, in this case, further emphasized by the volatility within the fog environment [3].

Internet of Things (IoT) also implies a distributed architecture, where processing and storage are usually performed within the cloud environment [4]. However, the interaction with the physical world, which is the basis of IoT scenarios, is executed in most cases across the targeted local domains where fog processing could be applied to enhance the overall efficiency of service performance. Still, to efficiently achieve this goal, it was necessary to overcome the heterogeneity of devices within the local environments, which was enabled by the recent popularization of the microservice architecture combined with the container-based virtualization [5]. This design approach has made the components of IoT services more isolated and portable, which was the essential factor that enabled their execution across heterogeneous devices, and ultimately, the implementation of the efficient fog processing layer [6].

The technologies mentioned above have initiated the transition within the fog computing research from finding a way of its efficient inclusion to the cloud processing plane towards investigating the problem of scheduling service components across the distributed execution environment [7]. As fog computing widens the processing plane to different execution environments, the challenge now is to detect the most appropriate place where a particular service component should be deployed to deliver the best quality of service. Across the scientific literature, different algorithms that tackle this problem can be found, and the majority of them utilize Kubernetes as the operational base of their approaches [8,9]. Kubernetes is an open-source container-orchestration tool intended primarily for the utilization within the cluster environment, and therefore, all nodes that make up the execution environment should be publicly available to communicate with the control plane [10]. However, we wanted to enable the efficient utilization of user devices for fog processing that are usually not publicly available since they are hidden behind routers with NAT or firewall, and their IP addresses and port mappings are unpredictable [11]. Thus, we did not utilize any existing container-orchestration tool in our approach, but our own custom scheduling system described in the continuation of this paper.

The main contribution of this paper is our scheduling algorithm that supports a dynamic execution dependent on the fluctuating network parameters and the pre-defined application QoS requirements. To verify its performance, we developed a distributed scheduling system that enables the inclusion of devices across private and public networks to the fog processing plane. In addition, we describe contexts relevant for the development of fog solutions within the IoT environment and the QoS parameters that evaluate their efficiency. Finally, the last contribution is the categorization of IoT services that affects the execution of our scheduling algorithm.

The remainder of the paper is structured as follows. Section 2 presents the related work. Section 3 describes the factors that affect the application of fog computing within the IoT environments. Section 4 introduces our scheduling system and the proposed scheduling algorithm, followed by Section 5, which displays the results of the conducted simulations that verify the targeted performance. Finally, Section 6 offers the discussion and conclusions gained from this research.

## 2. Related Work

Heterogeneous execution environments and the diversity of service scenarios are some of the basic characteristics of the IoT concept. The available computing resources differ across the entire IoT architecture, particularly within its lower layers, which include fog nodes and end-devices. Therefore, there are different options to determine the optimal location for specific service tasks’ execution. This section presents the recent research activities in the area of task scheduling in the fog computing environment, with a focus on dynamic rescheduling during the service runtime. Firstly, the directions in which the rescheduling (offloading) between nodes can be executed are listed. Secondly, different service models in the IoT environment that affect the offloading performance are presented. Finally, available mechanisms for task offloading are introduced.

### 2.1. Offloading in Fog Environment

Offloading within the fog-to-cloud environment can be initiated in different directions across the execution environment, depending on the application type and node capabilities [12].

The first option, which implies offloading from lower layers towards the fog nodes or the cloud, is triggered if the applications are already deployed as close as possible to data sources, but to the nodes that do not have enough resources to process all data. Consumer peripheral equipment in user-based scenarios, or machines in industrial use-cases, typically offloads the gathered data towards smartphones or fog nodes for storage and further processing. However, this processing can imply simple data reporting or more sophisticated data analysis that could cause the overloading of certain processing nodes. This is usually a consequence of constrained CPUs and the lower storage capacity that these processing devices possess [13,14]. In such cases, it is necessary to trigger offloading from nodes with insufficient resources towards the more powerful ones or the cloud.

Offloading between fog nodes usually occurs within load balancing and accessibility use-cases. In load-balancing scenarios, fog nodes executing a larger number of tasks offload a part of them to other fog nodes that are less occupied or more convenient in any aspect [15]. Within accessibility scenarios, information available to certain fog nodes has to be shared among additional fog nodes to enable the functionality of connected IoT devices [16]. Such situations typically occur when mobile IoT devices communicate with fog nodes. Therefore, when these IoT devices switch to the area covered by another fog node (e.g., a base station in a mobile network), their tasks should simultaneously migrate to the other fog node.

Offloading from the cloud towards the lower layers of the IoT architecture occurs for applications that need to be migrated closer to the edge of the network, mainly to meet the latency requirements [17]. For instance, within the traffic monitoring use-cases that do not require central processing, tasks are sometimes offloaded to roadside units closer to the vehicles to reduce the communication latency [18].

Finally, mutual offloading between the cloud and fog nodes is also an option, usually applied in cases where central processing is necessary, along with minimizing the execution latency [19]. In such cases, part of data analysis takes place on fog nodes, and the cloud processes fewer latency-critical data from multiple fog nodes (e.g., federated learning scenario). Additionally, this type of offloading is common when volatile environment parameters affect the optimal placement of particular service components. Hence, service components are migrated in both directions depending on the values of these parameters, which is also the idea of our approach described in this paper.

Described offloading strategies showcase different options of migrating task execution across the typical fog-to-cloud environment. However, the execution of task offloading in the reviewed literature usually considers an approach that implies forwarding the incoming requests among processing nodes. We believe that the utilization of service orchestration is more efficient to implement offloading because it eliminates, or at least reduces, the traffic generated by request forwarding, and it offers the option to process requests directly on the closest processing node. Thus, in this paper, we utilize the service orchestration approach to offload processing towards the fog environment to consequentially optimize service delivery in terms of targeted QoS parameters.

### 2.2. IoT Service Models

Different classifications of IoT services are available across the scientific literature, but their focus was usually to make a distinction between services that utilize different building blocks of the IoT architecture (sensing, data management, data processing, and execution) [20].

However, Gigli et al. [21] broke down services to the following four basic types according to their technical features: identity-related services, information aggregation services, collaborative-aware services, and ubiquitous services. Identity-related services include only IoT-enabled entity identification, upon which the data transmission is included within the information aggregation services. Collaborative-aware services add data processing on top of data collection services, and finally, ubiquitous services imply an interconnection between different collaborative-aware scenarios.

Insufficiently defined classifications in earlier phases of IoT concept development are probably caused by the lower number of available service scenarios. Thus, recent papers offer a more comprehensive overview of IoT service categories. Lee et al. define IoT services by their operative characteristics: sensing, data management, processing, and execution [20]. Each of these characteristics is determined by corresponding parameters that offer a sufficient number of values to describe most of the available IoT service scenarios. Services are then grouped into categories based on the similarity of these parameter values.

However, the goal of existing categorizations was usually to group the diverse IoT scenarios on their similarity or to enable service discovery and service recommendation [22,23]. Our motivation to categorize IoT services is to specify groups that determine the priority of QoS parameters that could be enhanced by applying fog computing. Thus, we had to define a new service categorization for our service scheduling algorithm, but since it also operates with distributed service components, we could partially utilize existing approaches that separately considered building blocks of IoT services.

### 2.3. Scheduling Execution and Offloading Algorithms

The first considerations of applying fog computing within the IoT architecture usually investigated the task-offloading strategies to determine the most appropriate node across the fog-to-cloud architecture that would process a particular task. There are various algorithms for task offloading within the existing scientific literature, but they usually include only one offloading scenario (e.g., IoT nodes to fog or cloud to fog) and base their decisions on a specific set of parameters. Wu et al. [24] provide a comprehensive categorization of such offloading decision schemes that includes Markov-based offloading decisions, graph-based offloading decisions, optimization-based offloading decisions, and deep learning-based offloading decisions. However, container-based virtualization enabled a way to overcome device heterogeneity in the fog environment and shift the research from offloading tasks between nodes to running collaborative distributed applications across the fog-to-cloud architecture.

Scheduling containerized service components has become a popular research subject since the emergence of orchestration tools, particularly the Kubernetes. In [9], Santos et al. present their network-aware scheduling approach for container-based applications in smart city deployments that was implemented as an extension to default scheduling mechanisms available in Kubernetes. The authors claim that their approach offers intelligent allocation decisions that provide proper resource provisioning in fog environments. The implementation of their algorithm implied labeling available nodes with different parameters, such as Round Trip Time (RTT), to enable the decision making to their “scheduler extender”, which is an extension of the kube-scheduler.

Caminero et al. present a similar approach in [8], where they propose a network-aware scheduling algorithm, which aims to select the fog node most suitable for the execution of an application within a given deadline. To implement network awareness, the authors utilized the iperf tool that calculates the network status. Thus, each worker node had the iperf-agent component running along with standard Kubernetes worker components. Additionally, the master node ran the iperf-server component that would periodically test each worker node and update its network performance metadata. Finally, pods had a defined deadline for their successful completion, and they were then scheduled based on the standard Kubernetes scheduling parameters and network metadata to meet this predefined deadline.

These existing approaches generally consider Kubernetes as their orchestrator for service scheduling across the fog-to-cloud execution environment. It is suitable because it is intended for utilization within the distributed cluster environment, and it provides the option to extend the default scheduler and consider the algorithm-specific procedures. However, it does not provide an optimal solution for its utilization within the fog-to-cloud architecture since it implies the connection of all included nodes to a single network. Thus, its utilization within the fog environment implies the overlay network where each fog node has to become a member of the simulated cluster [10]. Such an approach disables the direct communication between end-users and service components that could be scheduled within their local environments. This was the main motivation for the implementation of our scheduling system that overcomes these limitations by executing service scheduling across fog-to-cloud environments, including the fog devices in private networks that do not have static public IP addresses. The inclusion of such devices to processing infrastructure without overlay network is implemented by utilizing messaging system RabbitMQ as an interface to issue orchestrating commands towards fog devices within private networks. In continuation of this paper, we further explain the logic of our scheduling system and the proposed scheduling algorithms that enhance the QoS level in the delivery of IoT services.

## 3. Applying Fog Computing Concept within the IoT Architectures

The effectiveness of applying the fog computing layer within the existing IoT architectures depends on the objective of a specific IoT scenario. Each IoT service has its execution priorities and implementation restrictions that define the feasibility of applying fog computing principles within the specific scenario. However, the main goal in the design of the service architecture is to deliver its functionalities in the most efficient manner, and the efficiency of each specific service scenario is evaluated based on its primary goal. Within the IoT concept, fog computing primarily emerged as a response to the low latency requirements and the need to unburden the public network and cloud infrastructure of data traffic and processing load generated by IoT devices. Thus, the evaluation of the application of fog computing within IoT scenarios is primarily based on these premises and on all other consequential enhancements that can be achieved by shifting the processing load towards the network edge. One of those enhancements is the level of security in IoT architectures, which is often pointed out as the main obstacle towards further extensive growth of this concept. Employing local devices for raw data processing in local networks or applying security wrappers and cryptographic algorithms before forwarding data towards public networks could reduce the potential risk of service security breaches.

Achieving the full potential of fog computing within the specific IoT scenario requires that the primary goals, which determine its efficiency and could be affected by the utilization of fog computing, are first identified. The schedule of its service components along the fog-to-cloud environment is then tailored to improve the aimed efficiency based on the previously specified criteria.

Within this section, in Section 3.1, we first analyzed the main relevant contexts that define the feasibility and deployment restrictions for the utilization of fog computing within the specific IoT scenario. Then, in Section 3.2, we define relevant QoS parameters affected by the inclusion of fog computing in IoT environments. Finally, in Section 3.3, we describe a procedure of IoT service categorization that affects the application of service scheduling along fog-to-cloud architectures.

### 3.1. Contexts of Service Scheduling across Fog-to-Cloud Environment

The feasibility and the efficiency of service scheduling across fog-to-cloud environments depends on three relevant contexts:

- Available execution environment (device context);
- Specific service scenario (service context);
- Specific service user (user context).

The device context describes each fog device that offers its resources for the execution of service components. It is a prerequisite for the execution of service scheduling since the inclusion of fog layer depends on the available devices in local environments. The service context and user context are then utilized to determine the efficient schedule of service components along the fog-to-cloud continuum.

The device context is the description of the available fog processing nodes. The existing literature does not provide a clear and unified definition of a fog node, but numerous specifications similarly describe its functionality. The authors in [25] present their assumptions about the functionalities that fog devices should provide. These include computing, processing, and storing data in addition to routing and forwarding of data packets, sharing the network, computational, and storage load among themselves, and providing optimal support for the mobility of end-devices. However, more specific examples of device descriptions within fog environments are displayed in papers where practical experiments were conducted. Thus, the authors of [26] define fog devices with the following properties: storage capacity, processing capacity, memory capacity, number of processing units, and the service rate of one processing unit. These parameters define the storage and processing capabilities of a fog node that are significant for service scheduling. Hence, the scheduler can determine the most suitable fog node to run a specific service component based on this information. Therefore, we also consider the storage and processing capabilities of the fog node, along with other properties that in our approach affect the decision about the most appropriate location for the service deployment. We define the device context with the following properties:

- CPU (frequency, number of processing units);
- RAM (frequency, capacity);
- Storage memory (frequency, capacity);
- Location (network address - private and public IP, GPS location);
- Power supply (available battery capacity, AC power supply);
- Communication (capacity of IP connection, other supported protocols).

The service context defines the information about the specific service scenario. Although there are numerous different descriptions and classifications of IoT services [20,27,28], a unified definition does not exist. Still, the common baseline of existing definitions is that IoT services enable interaction with the physical world through sensor or actuator devices placed across targeted local environments. The goal of applying fog computing within IoT is to enhance service performance by migrating, at least partially, its processing components from the cloud towards these targeted local environments. However, each service scenario has different deployment limitations and operational goals that have to be considered to efficiently schedule its components across the fog-to-cloud continuum. Thus, we define the following properties that describe the service context within our approach:

- Data persistence (persistent/non-persistent);
- User reach (general/specific);
- Communication (IP connection capacity, other necessary communication protocols);
- Latency (maximum allowed latency);
- Security (low/high risk);
- Statefulness (stateful/stateless).

The user context provides the description of a specific service user. Most of the existing attempts to define the user context across the literature focus on the creation of accurate user profiles based on the relevant information about specific users [29,30]. Since our goal is to enhance the QoS in service delivery, we consider user inputs that affect the targeted QoS level as their relevant profiles. Additionally, as the authors in [31] point out, the main drawback of central cloud processing is the lack of location awareness in service delivery for mobile users. Thus, we consider the user’s network location as an important property that tackles this drawback within our service scheduling approach. Therefore, we define the following properties of user context:

- User location (network address, GPS location);
- Explicit limitations of QoS parameters.

### 3.2. QoS Parameters Affected by Applying Fog Computing

The quality of service implies relevant factors that evaluate the efficiency of service delivery. This subsection aims to identify QoS parameters of IoT services affected by the utilization of fog computing.

In the existing literature, the selection of relevant QoS parameters within the IoT domain is usually focused on a single layer of complete IoT architecture [32,33,34]. However, in [35], the authors present a comprehensive overview of QoS parameters across the overall IoT architecture, where the parameters are divided into three groups: communication, devices, and processing. The stated parameters are also applicable for evaluating fog computing within IoT environments. Still, in our approach, these parameters are considered mutually combined since the execution of a specific IoT service is evaluated as an integrated process from the user’s point of view. Thus, we define the following five QoS parameters affected by applying fog computing within IoT architectures:

- **Latency**: The most common parameter included in the evaluation of distributed computing systems. Total latency in a distributed system includes transmission delay (request sending time), propagation delay (data transition time), and operational delay (request processing time). Fog computing allows the execution of processing requests closer to end-users, or in the best-case scenario, within the same local environment. Thus, it reduces propagation and transmission delay, and consequentially the overall service response time calculated as the difference of the moment when the response is received ($t_{result}$) on the user’s side and the moment when the request is sent ($t_{request}$) towards the system:
  $$T_{total}=t_{result}-t_{request}$$
- **Network traffic reduction**: The amount of data traffic generated by IoT devices towards the public network has grown to the extent that it could cause unnecessary network congestion. Fog computing implicitly tackles this issue by reducing the volume of data that leave the local network. Placing fog nodes closer to the end-devices and utilizing them as data sinks or gateways for IP communication could reduce the overall amount of generated data traffic and improve service performance in the context of this QoS parameter. The quantification of this parameter is carried out by the amount of data traffic that is not generated towards the public network or by the number of locally processed requests;
- **Reliability**: The system’s reliability is its ability to process the incoming requests during a specific time duration. Fog nodes can be utilized to enable partial or complete processing of requests even in periods of network outages. Thus, fog nodes could buffer received requests until the network becomes operational again, or these requests could be fully processed within the local environment. Measuring the reliability from the user’s point of view is carried out by the following formula:
  $$R=\frac{\mathrm{number}\;\mathrm{of}\;\mathrm{successfully}\;\mathrm{received}\;\mathrm{responses}}{\mathrm{number}\;\mathrm{of}\;\mathrm{sent}\;\mathrm{requests}}$$
- **Scalability (throughput)**: Scalability is a property of computing systems that implies using the optimal number of processing resources necessary for efficient service delivery. Scalable architectures scale processing resources according to the volume of incoming requests to remain the constant throughput of the system. Fog computing introduces the option of horizontal system scaling that, besides increasing the system’s processing capabilities, also targets communication latency. Therefore, utilization of fog computing improves the system’s performance in the context of its throughput and latency, and thus, it ought to offer better results than cloud scaling strategies alone. Maintaining a constant throughput under various operational conditions is the best indicator of a scalable system and to measure the system’s scalability, we propose Little’s formula [36], where the throughput (*X*) is specified as the ratio between the average number of requests within the system (*N*) and the average time that each request spends in a system (*t*):
  $$X=\frac{N}{t}$$
- **Security**: The vulnerability of IoT devices [37], and the complete IoT system architectures, is often revealed as the major concern for the massive utilization of IoT. This is mainly the consequence of neglecting the security in the design phase of the solution development [38]. To change this course, OWASP (Open Web Application Security Project) initiated a separate IoT project designed to help manufacturers, developers, and consumers to better understand the security issues associated with the Internet of Things [39]. Within this project, OWASP has identified ten major security breaches of IoT that should be considered to achieve a sufficiently secure IoT system design. Additionally, these guidelines, combined with OWASP’s top ten application security risks [40], can be utilized to identify security breaches of the specific IoT architecture. After their identification, STRIDE method can be used to classify them, and afterward, DREAD method can be utilized to quantitatively assess the threat level of the evaluated system architecture [41]. However, we believe that prioritizing the local processing raises the overall service security level by default because the local deployment of at least one service component offers the opportunity to apply necessary security mechanisms before transmitting data to vulnerable public networks. Thus, we did not quantify the security threats in this paper, but we partially tackled this issue by prioritizing the local deployment in our scheduling algorithm.

### 3.3. Deployment Restrictions and the Categorization of IoT Services

This section describes our categorization of IoT services that determines the priority of identified QoS parameters and the deployment restrictions of a particular IoT scenario. Although there are numerous different IoT use-cases, each service scenario includes similar operational components from the following functional groups:

- Device interaction (sensor-data collection and actuator management);
- Different forms of data processing;
- Data storage.

These groups often imply separate microservices that together carry out the complete service functionality. However, the functional group of each microservice affects its deployment location across the distributed fog-to-cloud environment. Thus, to design an efficient scheduling algorithm, we first categorized IoT services and defined the deployment restrictions for specific use-cases.

The primary goal of our categorization is to determine the importance priority among previously identified QoS parameters for different categories of IoT services. Thus, the first step was to define service categories that briefly describe the essential purpose of a specific service scenario. Optimizations enabled by the application of fog computing are then mainly defined by these categories that primarily determine the goal of the specific service.

**Figure 1:**
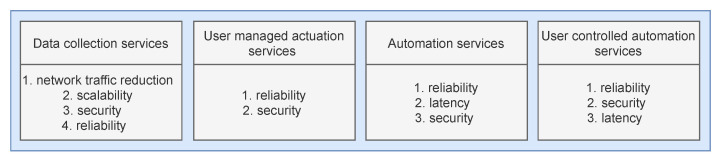
Prioritization of QoS parameters in different IoT service categories.

We define four different IoT service categories, as depicted in Figure 1, along with the associated QoS parameters ordered by their priority ranking within each category. These categories encompass most of the existing IoT scenarios described across the existing literature as the IoT scenarios with the highest application potential.

Data collection services and user-managed actuation services include basic scenarios, enabling simple IoT functionalities without intermediate data processing. Data collection services consider scenarios where the sensor data are gathered from sensor devices and stored within the cloud. Data are then offered directly to users or third-party companies for further processing (e.g., smart metering services). The high data volume generated in these scenarios is often pointed out as their most significant flaw, along with the security of transmitted data. Thus, fog computing optimizations within this category focus on reducing the data volume in public networks and strengthening the security of data transmission.

User-managed actuation services include basic scenarios where actuator control is enabled directly to users (e.g., door locks, heat control, etc.). Such services usually do not have a critical response time, but security and reliability are their essential properties since actuating devices perform actions that affect the physical world. Thus, applying fog computing in these scenarios should be pointed towards these QoS parameters.

The second two categories include more complex services that include all previously stated operational components. Automation services include completely autonomous scenarios, where actuation is executed autonomously based on the sensor data collected from the targeted environment (e.g., autonomous driving, automated parking ramps, etc.). Primary QoS parameters within such scenarios are reliability and latency, since actions must be completed almost instantly as a response to real-world situations. User-controlled automation services are a similar category where the actuation is to a certain extent controlled by the user (e.g., smart intercom, camera drones, etc.). Hence, latency in such scenarios is a less critical QoS parameter than reliability and security, since the threat of unauthorized actuation control exists within this category.

Another factor that affects the execution of our service scheduling are particular IoT services that require a specific deployment location across the distributed fog-to-cloud environment for their components. Service context parameters determine such deployment restrictions, and thus, they have to be considered before executing component scheduling. Our scheduling algorithm prioritizes local fog execution for each service component by default, so our goal was to detect use-cases that demand a different approach. Thus, if the service implies communication over constrained communication protocols, has high-security risk, or the specific user reach, its components should be explicitly deployed in a local environment, especially those intended to establish the interaction with the end devices. Additionally, the migration of stateful service components or those that include persistent data storage should be restricted since the effectiveness in such cases is lost because of the complexity and duration of their transfer. Therefore, service parameters: communication, security, user reach, data persistence, and statefulness, are considered first while executing our scheduling algorithm on the specific service component to recognize and address the described exceptional cases, if necessary.

### 3.4. Objective Function

In this subsection, we define the objective function of our scheduling algorithm that summarizes all previously described factors affecting the application of fog computing architecture. Applying fog computing benefits the IoT architecture in terms of the stated QoS parameters, as described previously in Section 3.2. Thus, our first goal is to prioritize the local execution of service components, as the local interaction between the user (*U*) and the service improves the level of each stated QoS parameter:

$$\Phi \left(sc_{i}\right)=max\left(locality^{fn_{k},U}\right)$$

The IoT service (*S*) is a set of *n* service components (*sc*):

$$sc_{i}\in S,\;i=1,...,n$$

and the set of *m* available fog devices (*F*) includes all nodes running in the cloud ($F_{c}$), user’s local network ($F_{l}$), and nodes in other private networks that are publicly exposed ($F_{p}$):

$$fn_{j}\in F,\;j=1,...,m\;\mathrm{and}\;F=F_{c}\cup F_{l}\cup F_{p}$$

while the ones considered in this step are the ones running in the user’s local environment:

$$fn_{k}\in F_{l},\;k=1,...,z\;and\;z<m$$

However, it is necessary to consider the possibility that each service component may have a deployment restriction that requires a specific execution environment as described in Section 3.3. Thus, we define the following two deployment restrictions:

$$\Phi \left(sc_{i}\right)\to fn_{j},\;fn_{j}\in F_{c}\;or\;fn_{j}\in F_{l}$$

If the defined objective function cannot schedule all service components to the user’s local environment (except the ones with deployment restrictions) as there may not be available local fog devices, we propose considering the latency as the second decision parameter since it also approximates locality. Thus, the second objective function aims to determine the available fog node that has the minimal latency in communication with the user requesting the service:

$$\Psi \left(sc_{i}\right)=min\left(latency^{fn_{h},U}\right)$$

but it only considers a subset of *s* publicly available fog nodes and the ones running in the cloud:

$$fn_{h}\in F_{c}\cup F_{p},\;h=1,...,s\;and\;s\leq m$$

The proposed approach favors service execution in fog environments to reach the improvements in terms of the stated QoS parameters, while the adjusted scheduling performance can be imposed by utilizing available deployment restrictions. In the following sections, we describe the algorithm based on the proposed objective function along with its implementation and performance evaluation.

## 4. Scheduling of IoT Services in Fog Computing Environment

IoT services usually include multiple components placed across a typical distributed architecture. Modern software architectural patterns also favor modular architectures due to the popularization of microservices and container-based virtualization (e.g., Docker). A combination of these two concepts could be the path towards the ultimate system development goal, which is to enable continuous delivery and high operational efficiency [42]. Container-based virtualization enables component isolation and enhanced component portability, which are necessary to achieve important system design imperatives such as scalability and reliability. However, the manual management of modular systems across distributed environments can become a complex procedure that is avoidable by utilizing the automated approach enabled by different orchestration tools (e.g., Kubernetes). These tools offer automated application deployment, scaling, and management. First, the administrator has to assemble the cluster environment and specify the execution parameters that define the desired application state. The tool then automatically schedules container execution across the available worker nodes in the cluster to first achieve and then to maintain the desired application state.

Since our goal is to schedule service components across the fog-to-cloud environment to improve the identified QoS parameters by applying our algorithm, we first examined the possibility of defining scheduling rules on top of the existing service orchestration tools. Fog computing implies the addition of computing resources to the cloud processing plane by including edge devices across local environments, which in most cases do not have static public IP addresses. The inclusion of such devices placed behind routers outside of the local master’s environment cannot be realized by default because all cluster nodes should be reachable to establish a functional Kubernetes cluster. Thus, the only option to organize a Kubernetes cluster that includes fog devices without static public IP addresses is to build a VPN that includes all entangled entities (master node and each device that joins the cluster as a worker node) since the Kubernetes master component kube-apiserver should be able to establish communication with the worker’s kubelet component [43,44,45]. However, this approach is not suitable in our use-case since the VPN creates the additional overhead along with Kubernetes components that is not desirable because of the limited resources of fog devices. Additionally, the utilization of a VPN could lower the reliability and latency because of the communication traffic routing through the VPN server.

Therefore, we could not implement our scheduling procedures on top of Kubernetes, so we examined available tools intended primarily for the implementation of the fog execution environment. We considered the KubeEdge and ioFog platforms, and both of these did not meet the requirements that were necessary to build our solution on top of them. KubeEdge runs on top of Kubernetes and its installation procedure is complex due to insufficiently detailed instructions. The deficiency of ioFog platform is its inability to support automated migration based on the environment parameters and specific rules of custom scheduling algorithms. Additionally, implementing plugins that target our specific purpose on top of these solutions was inefficient because of their complex functionality. Thus, we designed and implemented our scheduling system presented in Section 4.1 that considers previously specified contexts and service categorization to migrate service components based on our scheduling algorithm described in Section 4.2.

### 4.1. Formal Specification of Our Service Scheduling System

**Figure 2:**
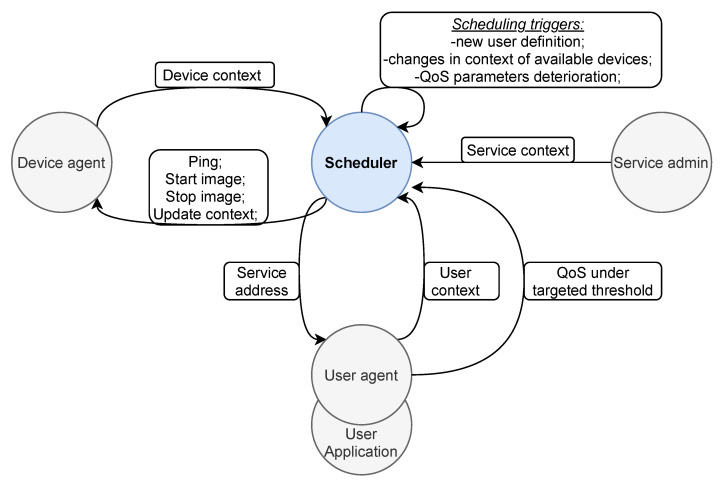
High-level formal model of the scheduling environment.

To implement our scheduling system, we had to model and deliver each of the described contexts in Section 3.1 to the **Scheduler**, a component that executes the scheduling algorithm. The system design model of our service scheduling system is depicted in Figure 2.

**Figure 3:**
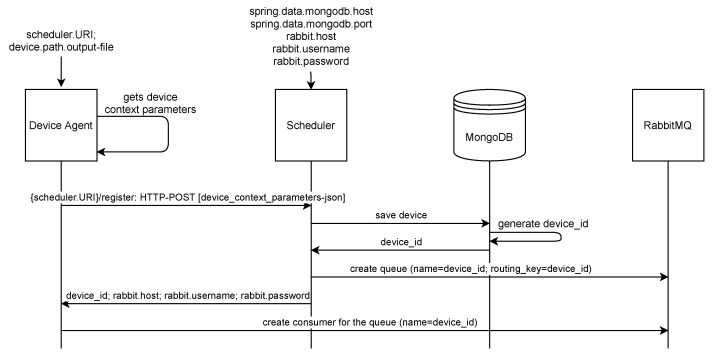
Device registration procedure.

Each device that joins our system as the processing fog node has to run the **Device agent** component that handles all necessary interaction with the Scheduler and executes the received commands. To join the execution environment, the Device agent sends the initial registration request containing device context information to the Scheduler (Figure 3). After its successful registration, the Device agent becomes available to the Scheduler that can send four different requests towards the device: ping request, request to start or stop the execution of a specific service component, or request to update its device context parameters.

**User agent** component runs on the user’s end-device along with the user application, and it also interacts with the Scheduler. Its purpose is to send the initial request containing user context information to the Scheduler, upon which it executes the scheduling procedure and returns a response containing the address of the targeted service component. Thus, the user application communicates directly with the requested service running on the fog node with the specified address. Additionally, if targeted user-specific limitations on QoS parameters are not satisfied, the User agent notifies the Scheduler with the request to execute a re-scheduling procedure.

The last entity in the depicted model is the service administrator that specifies the service context of a particular IoT service and its components. This information determines the category and deployment restrictions from Section 3.3 of the specific service scenario, and consequentially the course of applying our service scheduling algorithm.

**Figure 4:**
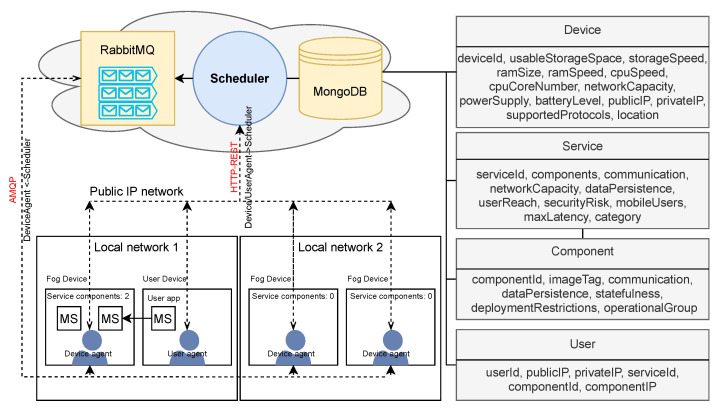
System architecture and the utilized data model.

The technical architecture of our scheduling environment is depicted in Figure 4. The Scheduler component has a public IP address and it is reachable to all other system components (Device agent and User agent) through its publicly exposed REST API. At startup, the Scheduler receives the input parameters that specify RabbitMQ and MongoDB connection details. All received context information of involved entities is stored within the MongoDB database, utilizing the data model depicted in Figure 4. The Scheduler utilizes the communication over the RabbitMQ message-broker to communicate with Device agents that are usually not reachable through the public network. Thus, the Scheduler creates a message queue within the broker for each registered device upon receiving its registration request. As a response to the registration request, the Device agent receives the parameters necessary to establish the connection with the RabbitMQ message-broker and the generated *device_id*. This *device_id* is also the name of the queue where the Scheduler sends commands for this specific Device agent, and thus, the local nodes are available to the Scheduler as they are subscribed through long-lasting connections to their corresponding queues within the RabbitMQ broker (Figure 3). It is also important to emphasize that we implemented synchronous communication through the RabbitMQ message broker to confirm the successful command execution and recognize if a particular fog node has become unavailable. The User agent also communicates with the Scheduler using the HTTP protocol, and the final communication between the user application and the requested service is direct once the service has been deployed to the selected fog node, as depicted in the lower left corner of the Figure 4.

In addition to communication with the Scheduler, the Device agent implements the retrieval of all device context information and running or stopping a specific service component that has to be accessible as a docker image. Utilizing Docker enables our system to support various platforms, which is important to overcome the heterogeneity of fog devices forming the execution infrastructure. If the specified service component image does not exist on a specific device, the Device agent downloads it, and starts (or stops) the specific container by issuing commands directly to the docker daemon. However, since IoT services imply multiple components migrated across distributed execution environment, the Scheduler keeps track of the location where each service container is running to provide the service discovery for collaborating components.

### 4.2. Algorithm for Dynamic Scheduling of IoT Service Components

The goal of our scheduling algorithm is to determine the most efficient deployment location for each service component along the fog-to-cloud continuum. It is necessary to execute different procedures to achieve this in such a volatile execution environment while supporting user mobility and previously described dynamic QoS parameters. Execution order of the procedures that implement our scheduling algorithm is defined in Algorithm 1, and their logic is described in Section 4.2.1–Section 4.2.4. In Section 4.2.5, we present the algorithm that enables the support for dynamic execution of our scheduling algorithm based on the considered QoS parameters.
**Algorithm** **1** Scheduling algorithm**Input:** 
service context, device context, user context1:serviceComponentsToSchedule = service.getServiceComponents2:Set deployment restrictions (service context, serviceComponentsToSchedule)3:Categorize available fog devices to local and public (user context, device context)4:Scheduling procedure-first stage (serviceComponentsToSchedule, device context)5:**if** (serviceComponentsToSchedule.notEmpty) **then**6:    Scheduling procedure-second stage (serviceComponentsToSchedule, device context)7:**end if**

#### 4.2.1. Setting Up Service Deployment Restrictions

The first procedure (Algorithm 2) considers deployment restrictions of service components based on the service context parameters. The significance of this factor has been aforementioned at the end of Section 3.3, and the goal of this procedure is to designate if a particular service component has to be scheduled to the specific environment. The procedure is triggered when the service administrator sends a service registration request, along with its context and service components description. Based on the received context parameters, the following three deployment restrictions can be defined as shown in the specification of Algorithm 2:

- Mandatory local execution (lines 2–3)—for service components (*SC*) intended to realize interaction with end-devices in service scenarios (*S*) that imply high-security risk (these components could be utilized to apply security mechanisms before data transmission to the public network), and for service components requiring specific constrained communication protocols (their reach is usually limited to the local environment);
- Mandatory cloud execution (lines 4–5)—for service components that are stateful or have persistent data storage, and are intended for mobile users since their frequent migrations could be more complex and time-consuming than acquired performance improvement;
- Preferred energy-supplied fog nodes (lines 6–7)—for service components that are stateful or have persistent data storage, and are intended for static users since such devices should provide a sufficiently stable execution environment due to their constant energy supply.

**Algorithm** **2** Set deployment restrictions**Input:** 
service context, serviceComponentsToSchedule**Output:** 
serviceComponentsToSchedule with deployment restrictions1:**for each** (SC in serviceComponentsToSchedule) **do**2:    **if** (SC.otherProtocolsRequired OR (S.highSecurityRequired AND SC.getType == device_interaction)) **then**3:        SC.setHardDeploymentRestriction(**local**)4:    **else if** (S.hasMobileUsers AND (SC.isStateful OR SC.hasPersistentData)) **then**5:        SC.setHardDeploymentRestriction(**cloud**)6:    **else if** (S.hasStaticUsers AND (SC.isStateful OR SC.hasPersistentData)) **then**7:        SC.setSoftDeploymentRestriction(**device.powerSupply==AC**)8:    **else**9:        no deployment restrictions10:    **end if**11:**end for**

#### 4.2.2. Device Agents Categorisation

The device agent categorization procedure (Algorithm 3) runs upon receiving the user registration request from User agent that specifies the required service and the targeted local environment, which is an important factor for designating the appropriate execution schedule. For each available Device agent, this procedure determines if it runs on a device placed within the same local network as the user (lines 4–5) or on a publicly available device outside the user’s network (lines 6–7). Device agents that do not meet any of these two criteria are ignored within the current iteration of scheduling algorithm execution since those fog nodes are unreachable to the user that requests the service.
**Algorithm** **3** Categorise available fog devices to local and public**Input:** 
user context, device context, availableFogDevices**Output:** 
two groups of availableFogDevices (local and public)1:**for each** (fogDevice in availableFogDevices) **do**2:    **if** (fogDevice.unavailable) **then**3:        availableFogDevices.remove(fogDevice)4:    **else if** (fogDevice.publicIP == user.publicIP) **then**5:        FogDevicesLocal⇐fogDevice6:    **else if** (fogDevice.publiclyReachableFlag) **then**7:        FogDevicesPublic⇐fogDevice8:    **end if**9:**end for**

#### 4.2.3. Scheduling Procedure (First Stage)

In the first stage of our scheduling procedure (Algorithm 4), the Scheduler considers running the components of the requested service in the local and cloud environment, based on the previously specified deployment restrictions (lines 2–6). The procedure cannot be successfully executed if the Scheduler cannot satisfy a particular hard deployment restriction that requires running a service component in the specific execution environment. In such cases, the procedure is terminated, and the user is notified of the unsuccessful service scheduling. The reason for such behavior is the assumption that if the Scheduler cannot satisfy mandatory deployment restriction of a particular service component, the service cannot offer its functionalities properly, and thus, it is not started at all.
**Algorithm** **4** Scheduling procedure (first stage)**Input:** 
serviceComponentsToSchedule, device context**Output:** 
deployed serviceComponentsToSchedule1:**for each** (serviceComponent in serviceComponentsToSchedule) **do**2:    **if** (serviceComponent.hardExecutionEnvironment == local) **then**3:        LocalFogDevices⇐serviceComponent4:    **else if** (serviceComponent.hardExecutionEnvironment == cloud) **then**5:        Cloud⇐serviceComponent6:    **else**7:        try: LocalFogDevices⇐serviceComponent8:    **end if**9:**end for**

Service components without mandatory deployment restrictions are also scheduled in this stage of our scheduling procedure to available devices in the local environment (line 7), preferably to those that are not battery powered if the specific service component has this soft deployment restriction. Thus, at the end of this stage of the scheduling procedure, service components are scheduled within the local environment and the cloud. If all components of the requested service are successfully scheduled in this initial stage of our scheduling procedure, the scheduling is completed and the User agent is notified about the IP address where the requested service is reachable.

#### 4.2.4. Scheduling Procedure (Second Stage)

The second stage of our scheduling procedure (Algorithm 5) is triggered if there are still service components left unscheduled after the completion of its first stage. In such instances, the Scheduler returns the registration response to the User agent containing the addresses of all publicly available fog nodes (line 2). Upon receiving this response, the procedure that measures the duration of ping request towards each available fog node is started by the User agent (lines 3–9). The results of this ping procedure are sent back to the Scheduler (line 10), which continues the execution of our scheduling procedure based on the received information. All publicly available fog nodes are sorted on the duration of their ping latency (line 12) to deploy the remaining unscheduled components on these nodes in the corresponding order (lines 13–15). Cloud deployment is also included within this stage of the scheduling procedure since publicly available fog nodes also include those running in the cloud environment. Thus, all service components should be successfully scheduled upon executing this stage of our scheduling procedure, and in that case, the User agent is notified about the IP address where the required service is reachable. However, if all service components are not successfully scheduled, the User agent receives a response stating the unavailability of the service due to the unsuccessful scheduling of all service components.
**Algorithm** **5** Scheduling procedure (second stage)**Input:** 
serviceComponentsToSchedule, device context**Output:** 
deployed serviceComponentsToSchedule1:**if** (serviceComponentsToSchedule.isNotEmpty) **then**2:    $Scheduler\overset{send(FogDevicesPublic)}{\to }UserAgent$3:    **procedure** measure ping durations(FogDevicesPublic)                 ▹ runs on UserAgent4:        **for each** (fogDevice in FogDevicesPublic) **do**5:           t⇐fogDevice.pingDuration6:           **if** (pingLatencyThreshold.notDefined OR *t*<pingLatencyThreshold) **then**7:               addEntryToPingMap([fogDevice:t])8:           **end if**9:        **end for**10:        $UserAgent\overset{send(PingMap)}{\to }Scheduler$11:    **end procedure**12:    PingMap.sortOnPingDurationAscending13:    **for each** (serviceComponent in serviceComponentsToSchedule) **do**14:        **for each** (fogDevice in PingMap) **do**15:           try: fogDevice⇐serviceComponent16:        **end for**17:    **end for**18:**end if**

#### 4.2.5. Dynamic Scheduling Based on the QoS Parameters

The previously described scheduling procedure provides the improved service execution architecture, offering enhanced efficiency in context of QoS parameters described in Section 3.2. Our scheduling procedure prioritizes scheduling of service components to local environments and then towards other available fog nodes, based on their ping duration from the user that requires a particular service. Thus, the reduction in traffic generated in the public network is consequentially achieved since the service execution is partially migrated towards the local environment, or it can be easily achieved by applying minor adaptations to the application’s execution logic. The security is also significantly strengthened since most of the stated security issues in OWASP IoT top ten [39] are easier to handle if the service component that interacts with the end-device is executed locally. The fog node where this service component is placed can then be utilized to implement necessary security procedures that would enable the realization of the system where privacy, device management, and data transfer are at the sufficient security level. Based on these premises, we can conclude that our scheduling procedure ensures the execution architecture that offers improved security and network traffic reduction by default.

The other three considered QoS parameters (latency, reliability, and scalability) are also initially tackled by the presented procedure, but because of the volatile execution environment and user mobility, their values are constantly fluctuating. Thus, to maintain the desired QoS level in terms of these parameters, it is necessary to enable on-demand execution of the scheduling algorithm, triggered when observed QoS values deteriorate (Algorithm 6). The crucial challenge is to recognize the necessity to re-run the scheduling procedure or replicate specific service components that may have become a bottleneck which has caused a drop in service performance. To achieve this goal, it is primarily required to observe the values of QoS parameters at the user side and execute the appropriate procedure upon their degradation.

The user’s application can track the values of latency and reliability parameters (lines 1–4 and line 18 in Algorithm 6), but the application alone cannot determine the throughput since it is the system attribute. Deterioration of latency could be a consequence of two different reasons: (1) increased service demand that causes processing congestion due to the growth of incoming requests and (2) changes in network throughput and latency between distributed components. The first reason actually refers to the service throughput, and therefore the user application can detect throughput degradation based on the observed latency. However, since the user application cannot certainly determine which of these two reasons was the actual cause of increased latency, it notifies the Scheduler through the corresponding User agent (line 5). The Scheduler then detects the real cause and resolves the problem. Upon receiving the latency downgrade notification, the Scheduler first verifies the CPU load on each fog node that runs the relevant service components (lines 7–12). If any of these Device agents reports CPU load above 90%, the Scheduler initiates the replication of service components running on the corresponding fog node (line 9). If no overloaded fog nodes are detected, the Scheduler stops all previously scheduled service components (line 14) and starts the execution of the scheduling algorithm from the beginning (line 15). The re-scheduling will not repeat the previous scheduling result as fog devices that caused the deterioration would not be considered in this iteration since their ping values in the repeated ping procedure would exceed the targeted latency limit.
**Algorithm** **6** Dynamic scheduling on QoS parameters**Input:** 
service context, user context, QoS constraints**Output:** 
replication or re-scheduling1:**for each**sentRequest**do**                                                                        ▹ runs on UserAgent2:    **if** (response.isReceived) **then**3:        latency = $t_{response}$ - $t_{request}$4:        **if** (latency > threshold) **then**5:           $UserAgent\overset{send(latency-downgrade)}{\to }Scheduler$6:           throughput⇐false                                                            ▹ runs on the Scheduler7:           **for each** fogNode in fogNodesExecutingServiceComponents **do**8:               **if** fogNode.getCurrentCpuLoad > 90% **then**9:                   fogNode.replicateServiceComponents10:                   throughput⇐true11:               **end if**12:           **end for**13:           **if** throughput.isFalse **then**14:               previouslyScheduledServiceComponents.stop15:               schedulingAlgorithm.execute16:           **end if**17:        **end if**18:    **else if** (response.notReceived AND reliability < threshold) **then**19:        $UserAgent\overset{send(reliability-downgrade)}{\to }Scheduler$20:        schedulingAlgorithm.execute21:    **end if**22:**end for**

The last considered QoS parameter is the reliability, calculated as described in Section 3.2. Thus, its degradation is detected when the application does not receive one or a certain percentage of responses from the required service (line 18). Although this may be caused by failure of a single component or user migration between different network environments, it is necessary to re-run the scheduling procedure again because the system must determine which fog nodes are the most suitable for the execution of service components under new conditions (line 20).

## 5. Simulation and Performance Evaluation

Since the functionality of the presented algorithms is highly dependent on network parameters, we had to utilize a tool that enables the manipulation of network parameters to simulate diverse network conditions and verify the expected behavior of our scheduling algorithm. IMUNES is an *Integrated Multiprotocol Network Emulator/Simulator* of IP-based networks [46] that enables the simulation of different network environments where virtual nodes can be linked either with other virtual nodes or with the physical network interfaces through simulated links [47,48,49]. These simulated links have different configuration properties, including bandwidth and latency that can be configured dynamically, which was the essential attribute for the execution of our simulation scenarios.

To utilize IMUNES we had to slightly adapt the logic of our scheduling system and adjust it to run jar modules instead of Docker containers since each node in IMUNES is in fact a Docker container, and it is not convenient to run another Docker container within an existing one. However, it is important to emphasize that the presented functionality, which includes running and stopping Docker containers across fog nodes, was first verified within a simple environment that included a single fog node and a cloud node to confirm the feasibility of the presented approach. After this confirmation, we implemented an adapted handler within the Device agent component that has the same logic as the one operating with Docker images, but instead, it operates with jar components. Thus, when the Scheduler sends a request to start a specific component to the Device agent, its adapted handler fetches the necessary jar file from the cloud (if it doesn’t exist on a specific fog node) instead of fetching it from the Docker hub, and then the pre-fetched jar file is started instead of Docker container.

**Figure 5:**
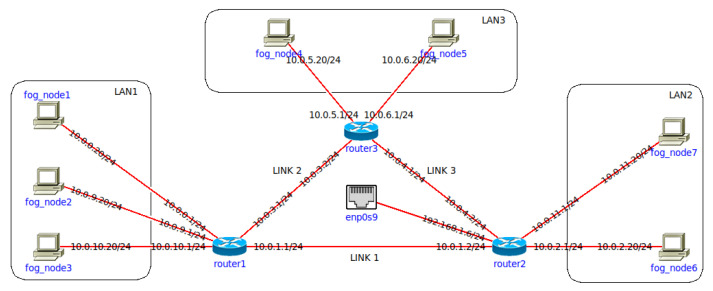
Simulation architecture in IMUNES.

We designed a network testbed depicted in Figure 5 to verify the expected behavior of the presented algorithm. It includes three separate LANs that are mutually connected through three different routers, and the entire testbed is linked to the public network over the external interface (*enp0s9*). Each depicted virtual *fog_node* is a docker container that includes Java, which enables us to primarily run Device agents and User agents, but also to schedule and start the necessary jar components of particular service. The Scheduler is placed on a server that simulates the cloud environment and it is reachable to all nodes within the simulation environment through the external interface. The idea of the designed testbed is to simulate three different LANs that could be anywhere in the global network, and the quality of communication between them would be affected by different factors that would cause constant fluctuations in terms of the examined QoS parameters. Although there would be a multi-hop distance between each of the depicted routers in a real-world scenario, a single link within our testbed is utilized to simulate different network conditions by applying dynamic adjustments to its configuration parameters (bandwidth and latency).

To compare the performance of our dynamic scheduling algorithm to the existing baselines, we implemented custom versions of our Scheduler component, one utilizing the default Kubernetes scheduling policy described in [50,51], and the other utilizing the Network Aware Scheduling algorithm (NAS) presented in [9]. Default kube-scheduler policy considers all available nodes and spreads the pods across available workers considering only CPU and RAM usage for each pod [51]. Thus, the custom Scheduler implementing the default Kubernetes scheduling policy considered only the stated CPU and memory usage of a fog node reported by the Device agent component to choose the most suitable node for service deployment. NAS was implemented on top of the default kube-scheduler policy since it considers the RTT parameter to prioritize the nodes remaining after the ones that could start the given pod are filtered out by the kube-scheduler. It also confirms that the chosen node has sufficient bandwidth to run the pod, but since all available nodes had sufficient and equal available bandwidth in our simulations, it does not affect the results. Thus, the custom Scheduler implementing the NAS algorithm considered ping-latency to the available fog nodes (RTT) to finally make a decision of the most suitable node for the service deployment.

Each simulation scenario described in continuation was executed first utilizing our Scheduler component, and afterward, its custom implementations utilizing the mentioned baseline approaches. The main limitation of the existing scheduling approaches is their inability to adapt the schedule depending on the volatile QoS parameters due to changeable network conditions as stated in [52]. Therefore, the goal of our simulations was to confirm the responsiveness of our dynamic scheduling algorithm to targeted QoS parameters and compare its performance to the existing approaches within the same environment. Thus, the expected results should showcase the performance improvement enabled by the utilization of our dynamic scheduling algorithm compared to the existing approaches due to its ability to re-schedule application components when the targeted QoS parameters deteriorate.

All simulations were conducted on the UBUNTU virtual machine running on a MacBook Pro device with a 2.5 GHz Dual-Core Intel Core i5 CPU and 16 GB of 1600 MHz RAM memory (8 GB was allocated to the virtual machine), while the simulated cloud environment where the Scheduler component, MongoDB, and RabbitMQ were running was a virtual machine with 2 vCPUs, 16 GB of RAM memory and Debian based OS (UBUNTU 18.04), on a server with 2.40 GHz Intel Xeon octa-core CPU (model E5-2630 v3).

### 5.1. Streaming Service Simulation Scenario

The first scenario was designed to verify the expected behavior of our scheduling algorithm and to confirm its ability to dynamically execute re-scheduling if the user-defined limits on the values of QoS parameters are not met. Data streaming is a common IoT service that is either a part of another larger service scenario or a stand-alone unit that implies the transmission and the reception of different kinds of data streams. Within IoT use-cases, end-devices often stream their sensing data towards intermediate data servers since their ability to store data locally is usually insufficient. To enable the efficient execution of such streaming scenarios, it is substantial to ensure the availability of a sufficient network bandwidth that is necessary to transport the transmitted data stream between the end-device and the server where the data are stored.

Thus, to simulate this scenario, we implemented a simple end-device application that generates data stream at a pre-defined transmission rate towards the reception service over the opened socket link. We also implemented a reception service that receives the incoming data and reports the number of received bits per second to the client. Exceptionally in this scenario, we enabled the information about the data throughput to the client, and it is affected only by network conditions since the server can absorb any volume of generated data. Consequentially, the User agent could explicitly request re-scheduling since the event of a fog node being overloaded is impossible in this scenario. The end-device application also defines the minimal throughput necessary for its functionality, and the goal of performed simulations was to verify that our system enables the appropriate deployment of the reception service to fulfill this condition.

#### 5.1.1. Test 1: Verification of Re-Scheduling Execution

In the first test, our goal was to confirm that the re-scheduling procedure was triggered at the right moment and that its execution properly migrates the service module to the most suitable fog node. The simulation included two fog nodes running the Device agent component (*fog_node6* and *fog_node5* in Figure 5), the Scheduler component running in the cloud, and the User agent component executed on the *fog_node3*.

At *t1* the User agent (*fog_node3*) sends a registration to the Scheduler that has two publicly available fog nodes at disposition (*fog_node5* and *fog_node6*), and the scheduling procedure is triggered. Since there are no available local fog nodes, the Scheduler requests ping duration values for each available fog node from the User agent requesting a service, to decide where to deploy components of the requested service. Table 1 shows the values of ping duration in moments when the scheduling procedure was triggered, and based on those values, the Scheduler would run the data reception service on the fog node that had the lowest ping duration. Datastream was generated at a speed of 66 Mbps, and the minimal throughput limit was set to 30 Mbps. Thus, to trigger the re-scheduling, we would lower the throughput to 30 Mbps on the link that connects the user node and a fog node where the data reception service is running at the given moment (Table 2).

**Figure 6:**
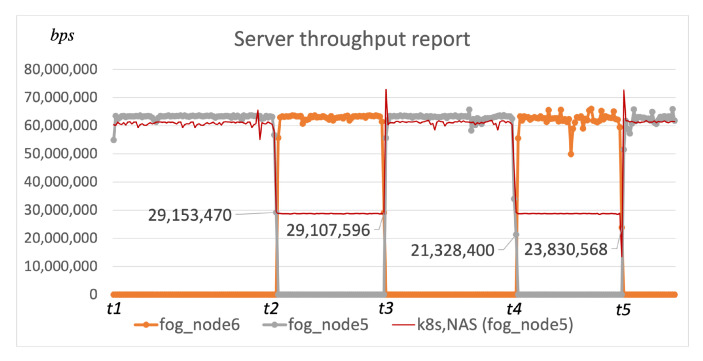
Throughput reported by the data reception service (traffic generation speed: 66 Mbps).

Figure 6 shows the throughput reported from the data reception component, and each thick-colored line represents a different fog node where this component was running. It can be seen that the re-scheduling was triggered instantly when the throughput value was under the pre-defined limit and that the service was then migrated to the more suitable fog node based on the ping value at the given moment. Kube-scheduler runs the scheduling procedure only when the application is not deployed, and it does not support the re-scheduling execution based on the fluctuating network QoS parameters. Since both custom Scheduler components are based on the Kubernetes platform, they had the same performance in this simulation scenario visualized with the thin red line in Figure 6. It can be seen that the re-scheduling procedure does not occur when the throughput decreases. Thus, it remains low, although another available fog node could satisfy the targeted QoS requirement.

#### 5.1.2. Test 2: Local Node Prioritization

**Figure 7:**
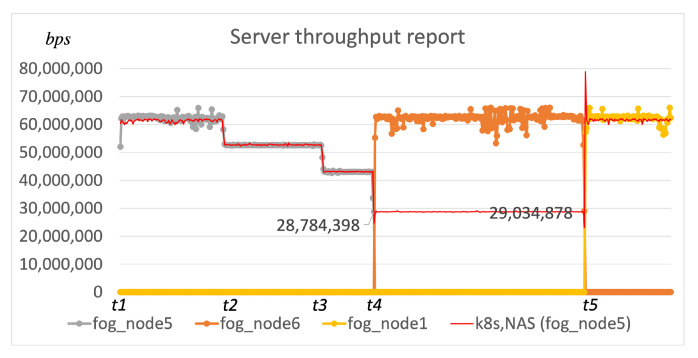
Throughput reported by the data reception service (traffic generation speed: 66 Mbps).

The second simulation included another fog node running the Device agent component (*fog_node1* in Figure 5) along with other entities from the previous simulation. The goal of our second test was to confirm that the local fog node is prioritized ahead of publicly available fog nodes in our scheduling procedure. Additionally, we adjusted the link throughput (*t*2, *t*3) as shown in Table 3 to confirm that the re-scheduling is not triggered when the throughput deteriorates, but only when the pre-defined limit is reached (*t*4, *t*5). Between *t*4 and *t*5 we added a local fog node (*fog_node1*) to the execution environment available to the Scheduler, and thus, in moment *t*5 the scheduling procedure did not require ping duration values from the user but it automatically started the required component on the fog node placed within the user’s local environment (Figure 7).

The thin red line shows the performance of custom Scheduler components utilizing the existing approaches based on Kubernetes. It is again confirmed that the re-scheduling does not occur when QoS deteriorates. Additionally, we increased the throughput on the *link2* at *t*5 to confirm that the local fog node is prioritized within our approach, although there is an available *fog_node5* that could satisfy the targeted throughput QoS limit. Consequentially, the performance from the moment *t*5 is the same as the performance utilizing our scheduling algorithm. However, the component has not been migrated towards the *fog_node1* within the user’s local environment since the re-scheduling does not occur and Kubernetes cannot schedule applications towards nodes without static IP address as described in Section 4.

### 5.2. Automation Service Scenario

Following the verification of algorithm behavior on a simple data-collection service scenario, we designed another simulation to confirm its performance on a more complex automation service category. The simulated service included three separate components: *receive* (reception of user requests), *processing* (simulated data processing), and *storage* (simulated database interaction). The simulated user application was implemented to constantly generate requests that were received by the *receive* component, then passed along to the *processing* component, and finally stored by the *storage* component, to measure the latency between each request and response. The goal of this simulation was to verify that our algorithm would successfully schedule a multi-component application and that it would properly handle its latency deterioration or outage of a certain fog node.

#### Automation Simulation Results

We conducted our automation service scenario at the same network testbed depicted in Figure 5 and the simulation environment included two publicly reachable fog nodes (*fog_node6* and *fog_node5*) running the Device agent component, the Scheduler component running in the cloud on the public network, and the User agent component executed on the *fog_node3*. Three service components (*receive*, *processing*, and *storage*) of the simulated automation service were registered so that the execution of Algorithm 2 would not assign any deployment restrictions to their service context. The acceptable latency limit was set to 5 s, and the request timeout was set to 10 s (the difference between latency and timeout values was made to obtain the latency values above the defined limit so they could be visible on the resulting graph).

**Figure 8:**
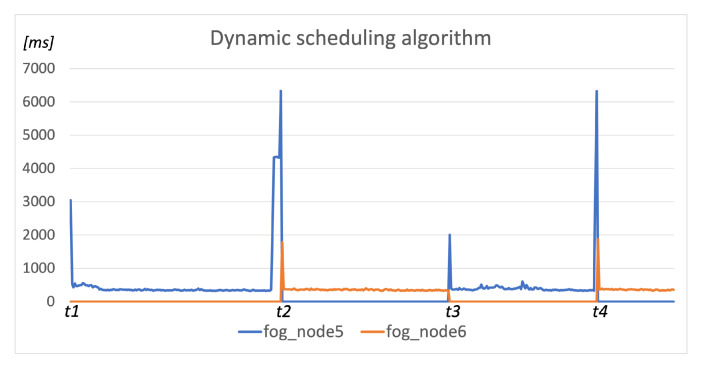
Request-response latency in automation service scenario simulation (dynamic scheduling algorithm).

Figure 8 presents the results of our automation scenario test execution utilizing the presented dynamic scheduling algorithm to deploy service components. The User agent and the user’s application were started on the *fog_node3*, and the first scheduling procedure executed at *t*1 placed all components on the *fog_node5* since it had lower ping-response latency than the *fog_node6*. The response latency was under the defined threshold until we increased the latency on *link2* to cause exceeding the pre-defined latency limit at *t*2. This initiated the re-scheduling procedure that migrated components to the *fog_node6*. The response latency was then again under the defined threshold until *t*3, when we terminated the execution of *fog_node6* and caused the request timeout. The re-scheduling procedure was executed again and the components migrated to the *fog_node5* (latency on *link2* was reset between *t*2 and *t*3), where they were running until *t*4 when the latency limit was exceeded again. Between *t*3 and *t*4, we started the execution of *fog_node6* again, and thus, after the re-scheduling procedure at *t*4, the components migrated back to the *fog_node6*.

The considered three-component application would include three pods within the Kubernetes environment since components defined within the same pod would have to be executed on the same worker node [53], and we wanted to enable the distributed execution of application components. The default kube-scheduler policy considers only CPU and RAM usage for node prioritization while scheduling each pod. Thus, it would schedule the first pod to the *fog_node5* or *fog_node6* as they have the same amount of computing resources available. The second pod would be scheduled to the different node, as it would have less CPU/memory usage since it does not run any pods yet. Finally, the third component would be scheduled again to one of those two nodes, having less CPU/memory usage. The utilization of the NAS scheduling algorithm would schedule all pods to the fog node having the shortest RTT since this is the prioritized scheduling condition, as it is also the case within our approach. However, the re-scheduling would not be executed due to the increased latency on the chosen fog node since NAS verifies this condition only when the kube-scheduler runs its scheduling procedure.

**Figure 9:**
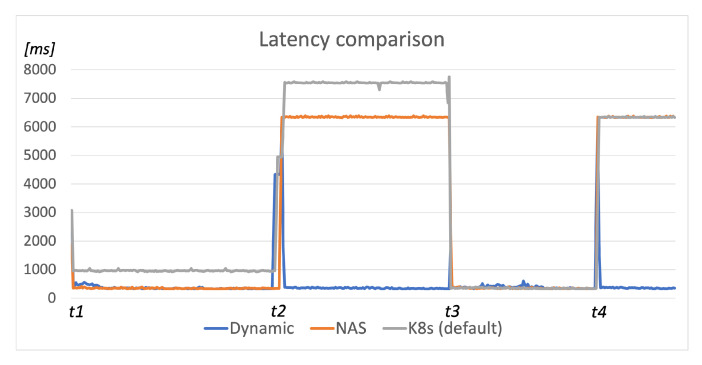
Latency comparison of different scheduling algorithms in automation service scenario simulation.

The automation simulation was repeated with custom Scheduler components utilizing the described scheduling policies to confirm the expected behavior, and the results are shown in Figure 9 along with the results of our dynamic scheduling policy. NAS has the same performance between *t*1 and *t*2 as our dynamic scheduling algorithm since it deploys all components of the service together on the node with the shortest RTT (*fog_node5*). The Kubernetes scheduler has deployed one component on the *fog_node6* and two components on the *fog_node5*, and because of their mutual communication through the *link3*, the latency is higher, which also confirms its less efficient performance in comparison to the NAS algorithm as described in [9]. At *t*2, the latency between the user and the *fog_node5* is increased, and our dynamic scheduling algorithm runs the re-scheduling procedure. However, since nodes are still running and the deployed application is available, the Kubernetes platform does not run the scheduling procedure as it does not recognize the latency deterioration. At *t3*, the *fog_node6* is terminated and the latency on the *fog_node5* is reset. Our scheduling algorithm migrates the application components due to the response timeout to the *fog_node5*, but the Kubernetes also recognizes the outage of the node where the pod was running. Thus, it also migrates the third component that was deployed on the *fog_node6* to the *fog_node5*. All three approaches have the same performance between *t*3 and *t*4, until we increased the latency again on the link towards the *fog_node5* and caused the migration of application components to *fog_node6* within our algorithm. The re-scheduling did not occur utilizing the other two approaches, so the components remained deployed on the *fog_node5* that had the increased latency.

## 6. Conclusions

This paper presents a comprehensive evaluation of the application of fog computing within a distributed IoT architecture. First, we analyzed the relevant contexts and categories of IoT services that affect the optimal schedule of service components across fog-to-cloud execution environments. Afterward, we briefly presented the first version of our distributed component scheduling system that includes agent modules for user and processing devices, while the central scheduling component manages the available entities and executes scheduling algorithms. The main contribution is our responsive scheduling algorithm that supports dynamic re-scheduling based on the fluctuating QoS parameters within the specific service scenario and enables the inclusion of local fog nodes to the processing plane.

The results of our simulations verified the performance of the presented algorithm and the functionality of our scheduling system. Additionally, we showcased the contribution of our responsive scheduling algorithm by confirming its performance improvement in comparison with existing scheduling approaches. Thus, we successfully implemented the algorithm that considers changes of volatile QoS parameters by executing the QoS level assessment from the perspective of the end-user consuming the requested service. However, this first version of our algorithm utilizes an individual approach for each user. In the future development, we plan to consider scheduling based on the user groups to optimize its efficiency by performing the scheduling procedure only if the requested components are not already running on nodes that provide the satisfying QoS level. Additionally, this version supports the dynamic execution of our algorithm, but the re-scheduling is always triggered by the user component (User agent). Thus, another optimization within our future work is to raise the responsiveness of our Scheduler component to context changes and execute re-scheduling that improves the overall QoS even if the existing schedule satisfies the requested user QoS limitations.

## Figures and Tables

**Table 1:** Ping duration values reported by User agent on fog_node3 (ms).

	*t*1	*t*2	*t*3	*t*4	*t*5
**fog_node5**	**544**	1393	**60**	1215	**71**
**fog_node6**	1568	**83**	1538	**58**	922

**Table 2:** Throughput on link1 and link2 (Mbps).

	*t*1	*t*2	*t*3	*t*4	*t*5
**LINK 1 (fog_node6)**	100	100	30	100	30
**LINK 2 (fog_node5)**	100	30	100	30	100

**Table 3:** Throughput on link1 and link2 (Mbps).

	*t*1	*t*2	*t*3	*t*4	*t*5
**LINK 1 (fog_node6)**	100	100	100	100	30
**LINK 2 (fog_node5)**	100	55	45	30	100

## Data Availability

The code of scheduling system components (Device Agent, Scheduler, and User Agent) and the presented simulation results are available at the following link: https://github.com/pKrivic23/FogResearch (accessed on 29 November 2021), and the IMUNES simulator is available at the following link: http://imunes.net/download (accessed on 29 November 2021).

## Abbreviations

The following abbreviations are used in this manuscript:

QoS	Quality of Service
IoT	Internet of Things
RTT	Round Trip Time
IP	Internet Protocol
VPN	Virtual Private Network
OWASP	Open Web Application Security Project
NAT	Network Address Translation
